# Pediatric acute disseminated encephalomyelitis following asymptomatic coronavirus disease 2019

**DOI:** 10.1016/j.idcr.2024.e01950

**Published:** 2024-04-17

**Authors:** Yuta Nakamura, Takahiro Namba, Yukiko Kawazu, Masato Yasui

**Affiliations:** Department of Pediatrics, Fukuyama City Hospital, Fukuyama, Japan

**Keywords:** Severe acute respiratory syndrome coronavirus 2, Coronavirus disease 2019, Acute disseminated encephalomyelitis, Myelin-oligodendrocyte glycoprotein antibody, Pediatric, Magnetic resonance imaging

## Abstract

After the severe acute respiratory syndrome coronavirus 2 (SARS-CoV-2) epidemic emerged, the virus spread rapidly worldwide, and outbreaks continued to occur intermittently. Here, we present the case of a 5-year-old boy with acute disseminated encephalomyelitis (ADEM) and initial symptoms of dysphoria and pain in the right lower extremity. Around the time of this episode, the patient exhibited no fever or respiratory symptoms. Brain magnetic resonance imaging (MRI) revealed multiple T2-weighted image/fluid-attenuated inversion recovery high-signal areas bilaterally subcortical to the deep white matter, corpus callosum, and bilateral basal ganglia. MRI of the cervical and thoracic regions indicated a long lesion with continuous T2WI high signal intensity in the central gray matter. Serum aquaporin-4 antibody and serum myelin oligodendrocyte glycoprotein antibody tests were negative and positive, respectively. A polymerase chain reaction test using nasopharyngeal swab fluid upon admission was positive for SARS-CoV-2. Patients with severe coronavirus disease 2019 (COVID-19) in the acute phase may show central nervous system symptoms. There have been no previous reports of ADEM in the subacute phase of COVID-19, lacking symptoms in the acute phase, as in the present case. Notably, ADEM can develop in the subacute phase of asymptomatic COVID-19 infection.

## Introduction

Acute disseminated encephalomyelitis (ADEM) involves the central nervous system (CNS) through a rapid autoimmune process and can occur following viral or bacterial infections or immunization [1], [2]. Since the beginning of the severe acute respiratory syndrome coronavirus 2 (SARS-CoV-2) and associated coronavirus disease 2019 (COVID-19) epidemic, several relationships between COVID-19 and ADEM have been reported [3], [4]; however, reports in children are scarce. In these reports, ADEM was reported to occur secondarily to severe symptoms in the acute or subacute phase, and non-neurological symptoms were often severe [3], [4], [5]. We describe the case of a 5-year-old boy who presented with neurological symptoms in the subacute phase of COVID-19 and was diagnosed with ADEM based on the magnetic resonance imaging (MRI) findings and positive myelin oligodendrocyte glycoprotein (MOG) antibodies despite the lack of acute COVID-19 symptoms.

## Case

A previously healthy 5-year-old boy presented with dysphoria beginning on day X-23. Prior to this onset, the patient was asymptomatic and exhibited no signs of fever. He had no history of illness and had received all vaccines mandated in Japan. Importantly, no vaccinations were administered during the last 12 months, including SARS-CoV-2 vaccines. On day X-17, the patient complained of right knee pain and numbness in the lower extremities and had difficulty walking. His symptoms fluctuated; however, he was evaluated at a pediatric clinic on day X-13 and was referred to our hospital on the same day. At the time of presentation to our hospital, no obvious abnormal findings were observed on physical examination. Thus, we referred to an orthopedic surgeon for a radiographic examination of right knee pain, which similarly resulted in no obvious abnormalities. Blood tests showed an elevated white blood cell (WBC) count of 16,820/µL. Furthermore, there was no elevation of inflammatory markers. When he returned to our hospital on day X-8, his gait disturbance had improved, and he was able to walk independently. Subsequently, his gait difficulty recurred, and he was unable to walk without support from either side. He began dropping objects that he was holding and could no longer use the spoon properly. The patient's lack of vigor gradually worsened; he became fatigued and spent an increasing amount of time lying in bed. The patient returned to our hospital on day X and was admitted. No headache, vertigo, diplopia, dysphagia, neck pain, or blurred vision was observed. Upon examination, the patient's blood pressure and pulse rate were measured at 91/68 mmHg and 115 beats/min, respectively. His temperature, respiratory rate, and oxygen saturation were 36.9 °C, 18 breaths per minute, and 98%, respectively. The patient was lethargic but obedient to simple verbal orders. No neck rigidity, Kernig's sign, or Burzynski's sign were observed. Deep tendon reflexes of the extremities were normal. Babinski's signs were absent, and his gait was unstable.

**Fig. 1:**
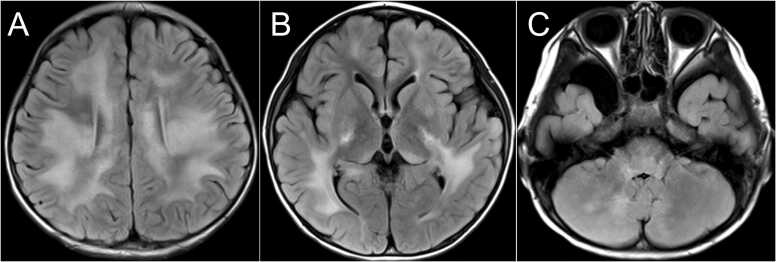
Magnetic resonance images (MRI) acquired using a 3.0-T system. (A) Level of cerebral cortex. (B) Level of basal ganglia and thalamus. (C) Level of pons. T2-weighted image (T2WI)/fluid-attenuated inversion recovery (FLAIR) axial image of the brain, showing high T2WI/FLAIR signal scattered in the bilateral subcortical to deep white matter, corpus callosum, bilateral basal ganglia and dorsal thalamus, bridge capsule, and bilateral cerebellar hemispheres. Swelling of the lesions was not evident. No hemorrhage accompanied the lesions.

**Fig. 2:**
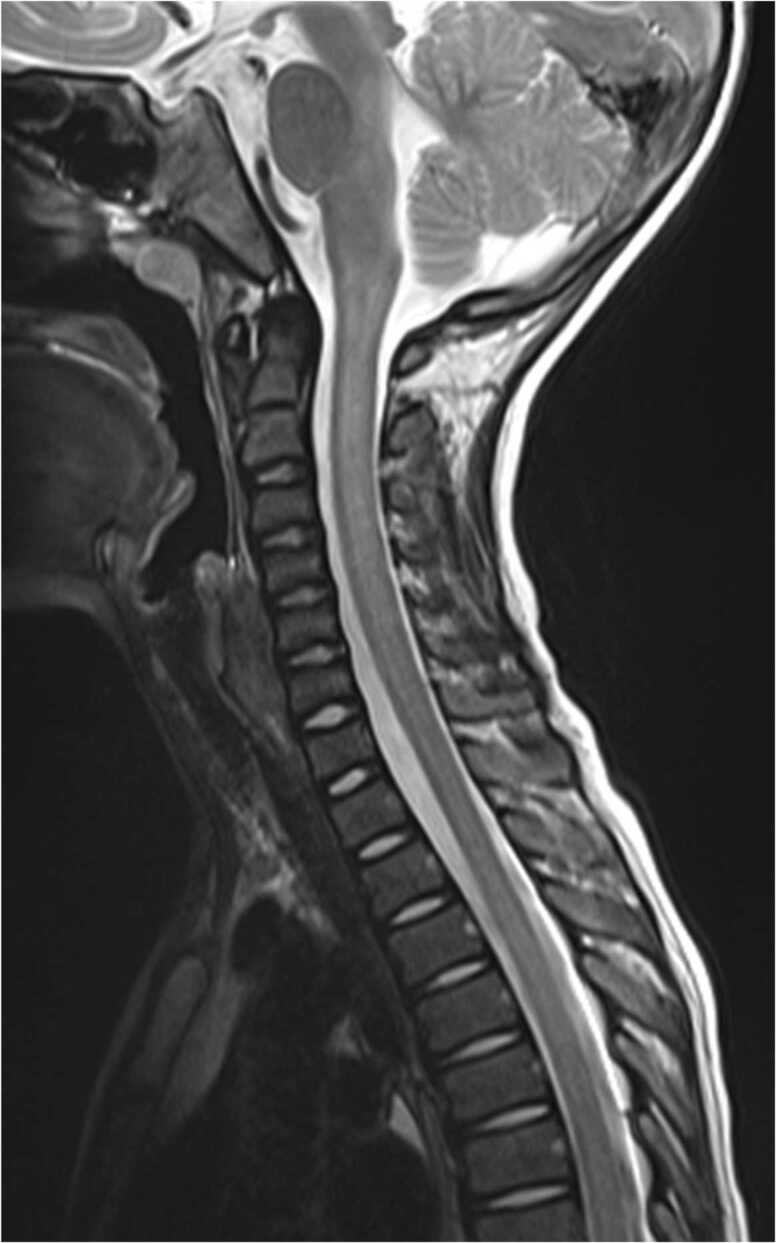
T2-weighted image (T2WI) sagittal image of the cervical and thoracic spine, showing T2WI hyperintense longitudinally extensive lesion in the central gray matter.

Brain MRI showed multiple T2-weighted image (T2WI)/ fluid-attenuated inversion recovery (FLAIR) high-signal areas in the bilateral subcortical to deep white matter, corpus callosum, bilateral basal ganglia, dorsal thalamus, bridge capsule, and bilateral cerebellar hemisphere. Swelling of the lesions was not evident. No hemorrhages were observed. In the cervicothoracic spinal cord, there was a long lesion with continuous T2WI high signal intensity in the central gray matter (Fig. 1, Fig. 2).

SARS-CoV-2 Polymerase Chain Reaction (PCR) test with nasopharyngeal swab fluid on admission was positive (Threshold Cycle, Ct value; 30.1). Notably, during the last 3 months prior to this episode, there had not been multiple outbreaks of SARS-CoV-2 infection around the patient or the patient's family. Despite attending group daycare, the child had not been in proximity to anyone with a confirmed SARS-CoV-2 infection for the past 3 months, leaving the source of the infection ambiguous.

Upon admission, laboratory findings indicated a WBC count of 11,600/µL, comprising 70% neutrophils, 25% lymphocytes, and 3% monocytes. Hemoglobin levels and platelet count were 13.6 g/dL and 47,900/µL, respectively. Blood chemistry revealed normal electrolyte levels and hepatic, renal, and thyroid functions. Coagulation function tests showed no abnormal values. The C-reactive protein and sedimentation rate were measured at 0.01 mg/dL and 10 mm/h, respectively. Notably, the complement system was elevated, with levels of C3, C4, and CH-50 recorded at 146 mg/dL (normal: 78–138 mg/dL), 46 mg/dL (normal: 11–31 mg/dL), and 50.5 U/mL (30–46 U/mL), respectively. A lumbar puncture revealed a cerebral fluid cell count of 44/µL, comprised of 85% mononuclear and 15% polymorphonuclear cells, and showed no red blood cells, with protein and glucose levels at 40 mg/dL and 54 mg/dL, respectively. Further, immunoglobulin G (IgG) level in the spinal fluid was elevated at 3.1 (normal: 1.0–3.0 mg/dL), and the IgG index was 0.83 (normal: 0–0.61). No oligoclonal bands were detected in the spinal fluid. Additionally, the serum MOG antibody test showed positive results, whereas the serum aquaporin-4 receptor antibody test showed negative results. The rheumatoid factor levels were not elevated at 3 IU/mL. Moreover, tests for antinuclear antibodies, anti-double stranded-DNA IgG antibodies, myeloperoxidase-anti-neutrophil cytoplasmic antibodies, and proteinase-3-anti-neutrophil cytoplasmic antibodies yielded negative results. Importantly, blood and spinal fluid cultures showed no bacterial growth, and Herpes virus PCR tests of the spinal fluid and stool virus isolation were also negative.

Therefore, the diagnosis was formulated based on the patient’s symptoms, history, MRI, and laboratory findings. The patient was treated with intravenous methylprednisolone (30 mg/kg/day) between days X + 1 and X + 3. The patient showed improvements in consciousness and walking disorders. Thereafter, prednisolone 2 mg/kg/day was administered. On day X + 6, the SARS-CoV-2 PCR test was repeated and confirmed to be negative. On day X + 7, the patient underwent an ophthalmologic examination, which revealed no optic neuritis or intraocular pressure abnormalities. A second course of methylprednisolone pulse therapy was administered from days X + 8 to X + 10 to further improve symptoms. Furthermore, prednisolone 1 mg/kg/day was administered starting on day X + 11. Brain MRI scan performed on day X + 13 showed a shrinking trend of T2WI/FLAIR high-signal areas in the subcortical and other regions, although there were some residual areas. The patient was discharged from the hospital on day X + 16, as he had completely recovered from gait disturbance and muscle weakness and was in good general condition. After discharge, there was no recurrence of ADEM. We were very careful in reducing the prednisolone dose, with careful attention paid to ADEM flare-ups. Brain MRI on day X + 100 revealed only a small lesion in the cerebral white matter. Prednisolone tapering was completed on day X + 180. Brain MRI on day X + 196 showed the disappearance of the abnormal lesions.

## Discussion

At the beginning of the COVID-19 pandemic, the primary symptoms were respiratory in nature [6]; however, it is now clear that COVID-19 affects a variety of organs, including the central nervous system and blood vessels throughout the body [7], [8]. The mechanism of COVID-19 neurological symptoms has been studied in two phases: acute and subacute [7], [8]. The immune-mediated responses during the subacute phases of SARS-CoV-2 infection might lead to the production of autoantibodies against neuronal antigens[7], [8], [9].

We diagnosed this case with ADEM according to the consensus criteria established by the International Pediatric Multiple Sclerosis Study Group [1]. ADEM is a multifocal and monophasic inflammatory demyelinating disease of the CNS that affects multiple areas of the brain and spinal cord white matter [1], [2], [10]. It is characterized by the acute onset of various neurological symptoms, including malaise, headache, nausea, and vomiting [1], [2], [10]. Moreover, these symptoms can escalate to respiratory failure and marked disturbances in consciousness due to brainstem lesions[1], [10]. ADEM occurs primarily in children, most commonly boys under 10 years of age, with a history of viral infection or vaccination within 30 days or earlier [1], [2], [10]. Notably, more than half of ADEM cases in children are likely to be positive for MOG antibodies, as in this case.

We also diagnosed this case as MOG-IgG-associated disorders (MOGAD) according to the diagnostic criteria proposed by the International MOGAD Panel [11]. MOG is a glycoprotein on the surface of oligodendrocytes [11] and plays an important role in CNS myelination. Prior infection may promote the subacute production of autoantibodies that cross MOG in molecular homology [11]. Subsequently, anti-MOG antibodies cause inflammatory demyelination through an immunogenic response [11], [12], [13]. Importantly, the association between SARS-CoV-2 infection and anti-MOG antibody production has been reported [12], [13]. In this case, the PCR test on admission showed a Ct value of 30.1, suggesting that at least 10 days had passed since the infection [14]. Six days of hospitalization later, the PCR test was repeated, and the results were confirmed negative. Therefore, we considered that this episode occurred in the subacute phase of SARS-CoV-2 infection. We speculate that SARS-CoV-2 infection may have triggered an autoimmune reaction that led to the production of MOG antibodies and the consequent development of ADEM.

In this case, the patient presented with dysphoria and numbness in the lower extremities without preceding fever or respiratory symptoms. There are few reports on ADEM associated with the onset of COVID-19 in children [15], [16], [17]. In many cases of ADEM with COVID-19, symptoms such as fever and respiratory symptoms preceding neurological symptoms have been observed [3], [4]. There have been no other reports of neurological symptoms as the first manifestation in the subacute phase without acute-phase fever or other symptoms, as in this case. Notably, the patient has not received any immunizations, including the SARS-CoV-2 vaccine, in the past 12 months. It is important to highlight that there have been some reports of ADEM after SARS-CoV-2 vaccination[18]. ADEM has been reported after many vaccinations, including influenza, Japanese encephalitis, measles, and mumps virus [19]. Epitopes shared between pathogen antigens and neuronal proteins may promote autoimmune responses through molecular mimicry [19]. In cases where preceding infection symptoms or recent vaccination events are present, ADEM could more readily be considered a differential. However, in the absence of such antecedents, it would be difficult to determine the possibility of ADEM. Early diagnosis and treatment initiation are important for the early recovery of patients with ADEM [2], [10]. Notably, the number of patients with MOGAD was reported to have increased after the COVID-19 pandemic [12]. As the SARS-CoV-2 epidemic persists, the number of infected patients will continue to increase, some of whom will have severe acute-phase symptoms, whereas others will have mild complaints. We should not overlook the possibility that ADEM may develop in the subacute phase of COVID-19, without acute symptoms.

The patient was treated with high-dose glucocorticoids promptly after diagnosis, and the treatment response was excellent. The first-line treatment for ADEM is high-dose glucocorticoids [2], [10]. Other immunotherapies, including intravenous immunoglobulin and plasma exchange, should be considered second-line therapies in aggressive cases, steroid non-responders, and relapsed cases [2], [10]. In this patient, the abnormal findings on brain MRI also improved in response to treatment. The oral steroid dosage was slowly tapered off, but no recurrence was observed. The patient is currently progressing on a monophasic course without sequelae. Despite limited reports on ADEM associated with COVID-19 in children, several cases have indicated possible sequelae or relapse [15], [20]. Prompt diagnosis and treatment initiation are desirable to avoid sequelae [2], [10]. Furthermore, careful oral steroid tapering and careful follow-up may be effective in preventing relapse [2], [10]. Prompt diagnosis is essential for obtaining a good therapeutic response as ADEM may develop even if acute phase symptoms are scarce in the preceding COVID-19. Many unanswered questions remain regarding the pathogenesis of ADEM caused by COVID-19, necessitating more reports and thorough research for clearer understanding.

## Ethics statement

Written informed consent for publication was obtained from the patient’s guardian.

## Funding

This research did not receive any specific grants from funding agencies in the public, commercial, or not-for-profit sectors.

## CRediT authorship contribution statement

**Masato Yasui:** Supervision, Writing – review & editing. **Yukiko Kawazu:** Writing – review & editing. **Takahiro Namba:** Conceptualization, Data curation, Writing – original draft, Writing – review & editing. **Yuta Nakamura:** Writing – original draft.

## Declaration of Competing Interest

None.
